# *Saccharomyces cerevisiae *chitin biosynthesis activation by N-acetylchitooses depends on size and structure of chito-oligosaccharides

**DOI:** 10.1186/1756-0500-4-454

**Published:** 2011-10-27

**Authors:** Hubert F Becker, Annie Piffeteau, Annie Thellend

**Affiliations:** 1 Laboratoire d'Optique et Biosciences, INSERM U696, CNRS UMR7645, Ecole Polytechnique, 91128 Palaiseau, France; 2 UPMC Univ Paris 06, CNRS UMR 7203, Laboratoire des Biomolécules, Paris 75005, France; 3 Université Paris-Diderot, Paris 75013, France

**Keywords:** chitin synthase, oligosaccharides, polysaccharide synthase, glycosyltransferase, polymerization activation

## Abstract

**Background:**

To explore chitin synthesis initiation, the effect of addition of exogenous oligosaccharides on *in vitro *chitin synthesis was studied. Oligosaccharides of various natures and lengths were added to a chitin synthase assay performed on a *Saccharomyces cerevisiae *membrane fraction.

**Findings:**

*N*-acetylchito-tetra, -penta and -octaoses resulted in 11 to 25% [^14^C]-GlcNAc incorporation into [^14^C]-chitin, corresponding to an increase in the initial velocity. The activation appeared specific to *N*-acetylchitooses as it was not observed with oligosaccharides in other series, such as beta-(1,4), beta-(1,3) or alpha-(1,6) glucooligosaccharides.

**Conclusions:**

The effect induced by the *N*-acetylchitooses was a saturable phenomenon and did not interfere with free GlcNAc and trypsin which are two known activators of yeast chitin synthase activity *in vitro*. The magnitude of the activation was dependent on both oligosaccharide concentration and oligosaccharide size.

## Background

Chitin, one of the essential fungal cell wall components, is a β-1,4 linked *N*-acetylglucosamine (GlcNAc) homopolymer. Since it is absent in plants and mammals and has an important structural role in the fungal cell wall, its biosynthesis is recognized as a valuable target for fungicides [1]. Chitin is synthesized by multiple membranous isoenzymes called chitin synthases (CHS) [2]. In *Saccharomyces cerevisiae*, the organism in which chitin biosynthesis has been most studied, three differentially expressed genes code for three different proteins belonging to the family of glycosyltransferases 2 [3,4]. CHS1p, is responsible for only 10% of the *in vivo *chitin pool, but accounts for most of the chitin synthase activity determined *in vitro *[4]. This activity is enhanced by trypsin and GlcNAc [2].

CHS are processive glycosyltransferases capable of successively transferring GlcNAc monomers from the UDP-GlcNAc donor to a growing polymer acceptor. Elongation takes place at the non reducing end of the polymer [5,6]. CHS isoenzymes belong to a subfamily of processive enzymes involved in polysaccharide biosynthesis such as cellulose and hyaluronan synthases [7,8]. The 3D-structure of a non-processive member of glycosyltransferases family 2 had shed some light on both their donor and acceptor sites [9]. In contrast, due to the lack of structures, donor and acceptor sites have not been delineated in the case of polysaccharide synthases. While the donor sites can be inferred from sequence comparison between processive and non-processives glycosyltransferases, the way polysaccharide synthases accommodate the growing polysaccharide is totally unknown.

As for any polymer, chitin formation is expected to require chain initiation, elongation and termination. Several mechanisms of polysaccharide priming by oligosaccharides, either in a free or conjugated form, have been described, specifically Carbohydrate polymerase GlfT2 from *Mycobacterium tuberculosis *acts on glycolipid acceptors for the galactan polymer synthesis [10]. Also, experiments on cellulose synthase from cotton fiber led to the proposal of a model in which cellulose synthesis would be initiated by a sitosterol-β-glucoside [11]. Important outcomes of this model were the fact that a certain number of discrete steps should precede the final elongation, and the existence of an oligoglucoside species as a discrete intermediate. Free glycoside utilisation, as primers, are described for other glycosyltransferases, such as β-D-xylosides used for glycosaminoglycans chain biosynthesis on proteoglycan core proteins [12]. Other polysaccharide synthases responsible for the addition of a sugar monomer to oligosaccharide acceptors have been described for hyaluronan [13], chondroitin sulfate [14], heparan sulfate [15], glycogen [16] and heparosan [17]. Also, high concentrations of GlcNAc were shown to prime *N*-acetylchitopentaose synthesis by the oligosaccharide synthase NodC [6] for the synthesis of rhizobial lipo-chitin oligosaccharides (Nod factors). Concerning chitin synthase, a priming by GlcNAc has never been observed and GlcNAc is thought to be an allosteric activator [18-20]. McMurrough and Bartnicky-Garcia tested a range of compounds structurally related to GlcNAc but none successfully substituted for GlcNAc in the allosteric activation of chitin synthesis, although *N*-acetylchitooses exerted a slight stimulation of substrate incorporation supposed to a different mechanism as GlcNAc [21].

In order to elucidate the role of oligosaccharides, a systematic study was carried out with preformed oligosaccharides on a chitin synthase assay on *S. cerevisiae *microsomal preparations. We used oligosaccharides of various degrees of polymerization (DP) and from different structures as well as *N*-acetylchitooses (β-(1,4) GlcNAc units) or β-(1,4), β-(1,3) or α-(1,6) glucooligosaccharides.

## Methods

All reagents were purchased from Sigma and Merck Biochemicals, unless indicated otherwise in the text. UDP-*N*-acetyl[^14^C]glucosamine was from NEN Life Sciences Products. *N*-acetylchitooses were either obtained from Dr R. Geremia (CERMAV Grenoble, France) or purchased from Sigma. Both batches were of identical purity (>95%).

### Preparation of membrane fractions

*Saccharomyces cerevisiae *cells (baker's yeast, 10 g) were washed and resuspended in 10 mL of 50 mM Tris-HCl (pH 7.4) - 2.5 mM MgCl_2_. After addition of glass beads (0.25-0.50 mm, 10 mL), cells were broken by shaking on a vortex for 7 cycles of 30 s alternated with 30 s cooling on ice. After decantation of the solution, the supernatant was recovered, centrifuged at 1000 × g for 10 min and then at 12 000 × g for 10 min to remove mitochondria. Membrane fractions were isolated from this extract by centrifugation at 100 000 × g for 1 h. The membrane pellet was resuspended in approximately 500 μl of 50 mM Tris-HCl (pH 7.4), 30% glycerol. Membrane preparations were conserved at -80 °C at protein concentration around 60 mg/ml, as determined by Bradford protein assay (Biorad) [22].

### Preparation of laminarin and dextran oligosaccharides

Laminarin and dextran (1 g of each) were dissolved in 10 ml 0.1 N HCl and acid hydrolysis performed at ebullition for 4 h. Mixtures were diluted with water, neutralized with Dowex 2 × 8-200 resin (hydroxyl charged) and lyophilized. Oligosaccharides with DP from 3 to 6 were obtained after Bio-gel P2 chromatography purification as previously described for *N*-acetylchitooses [23].

### Chitin synthase assay

Under our assay conditions, the main enzymatic activity is CHS1p activated by trypsin [4]. At 150 μg of *S. cerevisiae *membrane fractions containing chitin synthase activity a common standard reaction mix (in a total volume of 50 μl: 5 μl digitonin 2%, 5 μl magnesium acetate 50 mM, 5 μl trypsin 0,2 mg/mL (10000 units/mg protein, Sigma), 2 μl *N*-acetylglucosamine 1 M, 2.5 μl UDP-*N*-acetylglucosamine 10 mM, 1 μl UDP-*N*-acetyl[^14^C]glucosamine (10 nCi, 288 mCi/mmol) in Tris-HCl buffer 25 mM pH = 7.4) was added. Incubation was performed at 30°C for 10 min, the reaction was stopped with 1 ml of 10% trichloroacetic acid. The radioactivity in the insoluble fraction was counted after filtration through glass-fiber filter (Whatman, GF/C). The soluble fraction containing unincorporated UDP-*N*-acetyl[^14^C]glucosamine was discarded. The filter was washed three times with 1 ml of 1 M acetic acid/ethanol: 30/70 and once with 1 ml of 95% ethanol. The filter was transferred to a scintillation vial containing 4 ml of Optiphase Hisafe (Wallac) and the samples were counted in a scintillation counter (LKB 1214 RackBeta).

To determine the effect of adding different oligosaccharides chain lengths to the chitin synthase assay, membrane fractions were preincubated with either β-(1,3), β-(1,4) or α-(1,6) glucooligosaccharides or *N*-acetylchitoses (from bioses to octaoses) in 25 mM Tris-HCl pH 7.4 for 15 min at 30°C before addition of the common standard reaction mix. Oligosaccharides were used at a concentration of 1 mM unless otherwise stated.

### Chitin digestion by chitinase

[^14^C]-chitin, prepared as described above, was digested by *Streptomyces Griseus *chitinase (5.10^-3^U, 0.7 U/mg, Sigma) at 37°C for 24 h in phosphate buffer 2 mM pH = 6. Samples were boiled for 5 min and 1/5 of the sample was applied to TLC plates. Samples were analysed on precoated TLC plates silica gel 60, Merck. The chromatogram was developed with 1-propanol/water/ammonium hydroxide 30% (70:30:1.5, vol/vol/vol). Radioactive spots were detected by means of an automatic TLC-linear analyzer (LB 2821 Berthold).

### Effect of GlcNAc and trypsin

The *N*-acetylchitose activation effect was tested in absence, or at low (1 mM) GlcNAc concentration as described above. Alternatively, activation by trypsin was carried out before polymerization. In this case, membrane fractions were preincubated at 30°C with trypsin from bovine pancreas (1 μg, 10000 units/mg protein, Sigma) in 25 mM Tris-HCl pH 7.4. After 15 min, soybean trypsin inhibitor (2 μg, Sigma) was added and incubation followed for 15 min.

## Results and Discussion

### Catalytic activity of CHS

**Figure 1:**
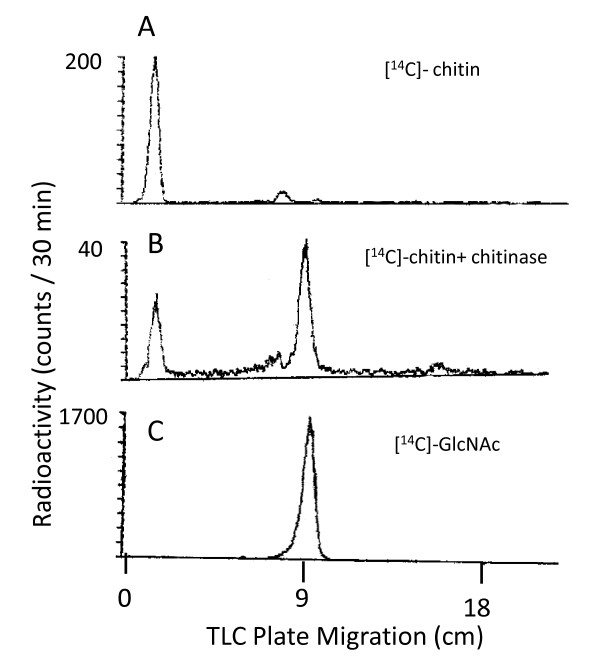
**Thin layer chromatography analysis of [^14^C]-chitin, produced *in vitro *by *S. cerevisiae *membrane fractionation**. The CHS reaction product was digested by *S. Griseus *chitinase and analysed on silica 60 TLC plates as described in Materials and Methods. Samples were loaded onto the TLC plate before (panel A) or after 24 h incubation with commercial chitinase (panel B). In panel C the reference sample containing commercial [^14^C]-GlcNAc was applied.

CHS activity assays were performed using *Saccharomyces cerevisiae *microsomal preparations by measuring the rate of formation of [^14^C]-chitin from UDP-*N*-acetyl[^14^C]glucosamine [4]. Oligosaccharides of different nature and length were preincubated with microsomes before the chitin synthase assay was performed. Unless otherwise stated, trypsin was routinely included as a required activator in the common reaction mix. The product of the reaction was unambiguously identified as chitin by specific digestion with commercial chitinase and chromatographic characterization of released GlcNAc units (Figure 1).

**Figure 2:**
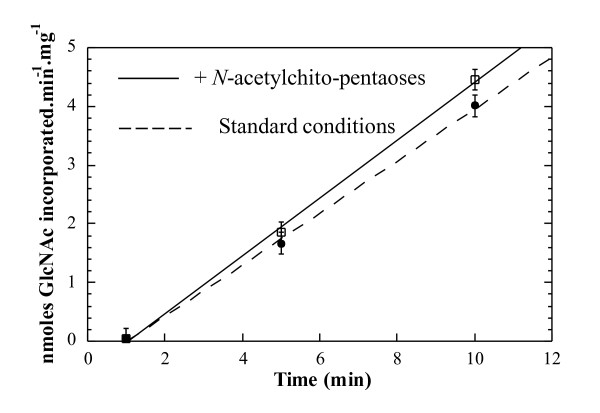
**Time course of chitin synthesis in presence or absence of *N*-acetylchitopentaoses**. Kinetics of two chitin synthase assays following preincubation with (open boxes, solid line) or without (black circles, dotted line) 1 mM *N*-acetylchitopentaoses. The linear part of the curve (up to 12 min) is shown.

The activating effect of *N*-acetylchitoses on chitin synthesis was studied over time with 0.5 mM UDP-GlcNAc and 1 mM *N*-acetylchitose concentrations. These concentrations are comparable to the UDP-GlcNAc Km of 0.25 mM displayed by yeast CHS 1 [4,24]. Initial linear portions of the kinetics obtained in presence and absence of *N*-acetylchitopentaoses were compared and initial velocity values were calculated (Figure 2). We obtained, 2.3 ± 0.1 nmol GlcNAc incorporated.min^-1^.mg protein^-1 ^in the presence, and 1.9 ± 0.09 nmol-GlcNAc incorporated.min^-1^.mg protein^-1 ^in the absence of *N*-acetylchitopentaoses. Thus, the initial velocity increase is about 20% and similar activation was observed for *N*-acetylchito -tetra or octaoses (Table 1).

**Table 1:** Per cent activation of [^14^C]-GlcNAc incorporation into chitin polymer in presence of oligosaccharides from different lengths and structures.

DP	*N*-acetyl-chitooses β-(1,4)-Glc-NAc	Laminari-oses β-(1,3)-Glc	Dextro-oses α-(1,6)-Glc	Cello-oses β-(1,4)-Glc
2	13,3% ± 2	2% ± 1,1	nd	nd
3	10% ± 1,5	1,6% ± 1,2	2% ± 1,3	nd
4	17,3% ± 0,8	0,5% ± 0,5	1,5% ± 1,2	nd
5	23,3% ± 1,1	1% ± 0,8	2,1% ± 1,5	3% ± 2
8	24,4% ± 1,2	nd	nd	nd

Chitin synthase activity of *S. cerevisiae *membrane fractions was measured as incorporation of [^14^C]-GlcNAc into a filter-retained insoluble material. Assays were performed upon preincubation of microsomes with *N*-acetylchitooses β-(1,4)-GlcNAc, laminarioses β-(1,3)-Glc, dextrooses α-(1,6)-Glc, or cellooses β-(1,4)-Glc. Length of oligosaccharides tested varied from bioses to octaoses (DP = degree of polymerization). Per cent activations were calculated compared to standard assay without added oligosaccharides. Results are the average of five independent experiments (±SD). Activation for some oligosaccharides was not determined (nd).

### Effect of oligosaccharide structure and length on chitin synthesis

*N*-acetylchito-bi and *-*trioses in the CHS test mixture had little effect on chitin synthesis (enhancement around 11%), and these results were poorly reproducible (Table 1). By contrast, *N*-acetylchito-tetra, *-*penta and *-*octaoses clearly stimulated GlcNAc incorporation from UDP-[^14^C]-GlcNAc into chitin, reaching up to 25% enhancement for the *N*-acetylchitooctaoses (Table 1). A clear-cut effect of the oligosaccharide size on the extent of activation was observed.

Oligosaccharides from other carbohydrate series such as laminari-(β-(1,3-gluco)), dextro-(α-(1,6-gluco))- or cello-(β-(1,4-gluco))-oligosaccharides did not stimulate [^14^C]-GlcNAc unit incorporation into [^14^C]-chitin (Table 1). The lack of effect of oligosaccharides from other series argues in favor of a specific effect of GlcNAc oligomers. Moreover, in order to achieve acceptable activation reproducibility, the *N*-acetylchitooses must be of at least 4 residues.

### Effect of chitooligosaccharides versus chitin synthase activators

**Figure 3:**
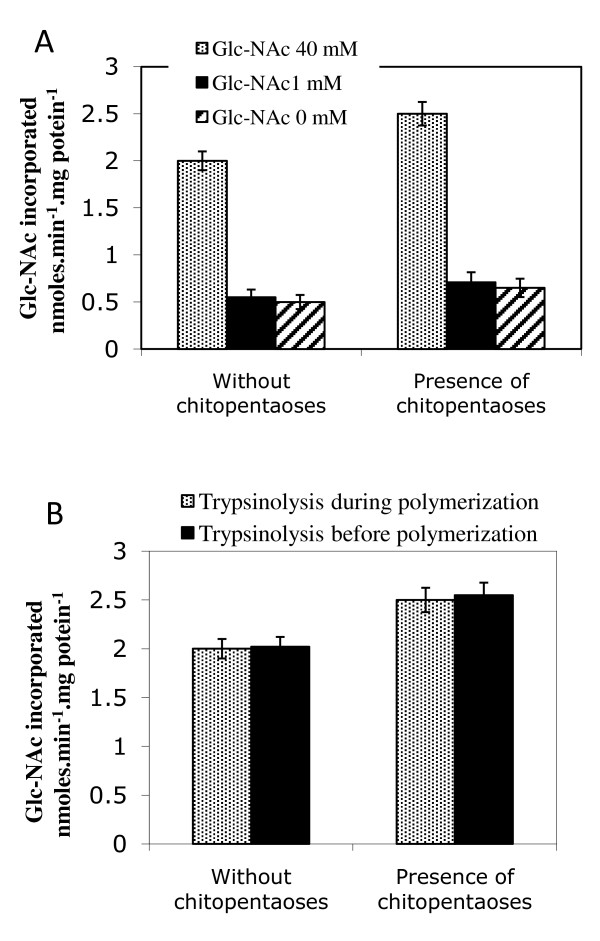
**Activation by *N*-acetylchitooses is independent on Glc-NAc and trypsin**. (A) [**^14^**C]-chitin was prepared and quantified as described, except that Glc-NAc was present at 40 mM (grey bars), 1 mM (black bars) or absent (hatched bars) in the incubation mixture. (B) [**^14^**C]-Glc-NAc incorporation into chitin measured after two different conditions of CHS activation by trypsin: during (standard conditions, grey bars), or before (black bars) chitin polymerization. Results in presence or absence of ***N***-acetylchitopentaoses are shown.

It is known that both trypsinolysis and a high (40 mM) GlcNAc concentration enhance *in vitro *chitin synthesis [25,19]. In order to test whether the activation on chitin synthesis by *N*-acetylchitooses could interfere with these two activators, we used these *N*-acetylchitooses as potent substitutes. The effect of 1 mM *N*-acetylchitopentaoses was measured in the absence of GlcNAC or at low (1 mM) and high (40 mM) GlcNAc concentration. Activation profiles, by *N*-acetylchitopentaoses, (~20%) were identical to that of standard assay (40 mM GlcNAc), but with a 3-fold reduction of UDP-GlcNAc incorporation (Figure 3A). Similar results were obtained with other *N*-acetylchitoose lengths (data not shown). One can thus conclude that the *N*-acetylchitooses did not substitute for GlcNAc. Both activating effects seem to be independent, suggesting that the two species bind to two different sites in a random manner.

Interference between the effect of *N*-acetylchitooses and activation by trypsin was also studied. In standard test conditions, microsomal preparations were activated by trypsin at the beginning of the reaction. In a parallel protocol, a preactivation of the microsomal preparation by trypsin was performed, then terminated by addition of trypsin inhibitor. Initial velocity in the presence of *N*-acetylchitooses clearly reached same value, whether or not trypsinolysis occured before or during chitin polymerization (Figure 3B). This result excluded a possible effect of chitin synthase protection by *N*-acetylchitooses against overdigestion by trypsin.

### Effect of oligosaccharide concentration on chitin synthase activity

**Figure 4:**
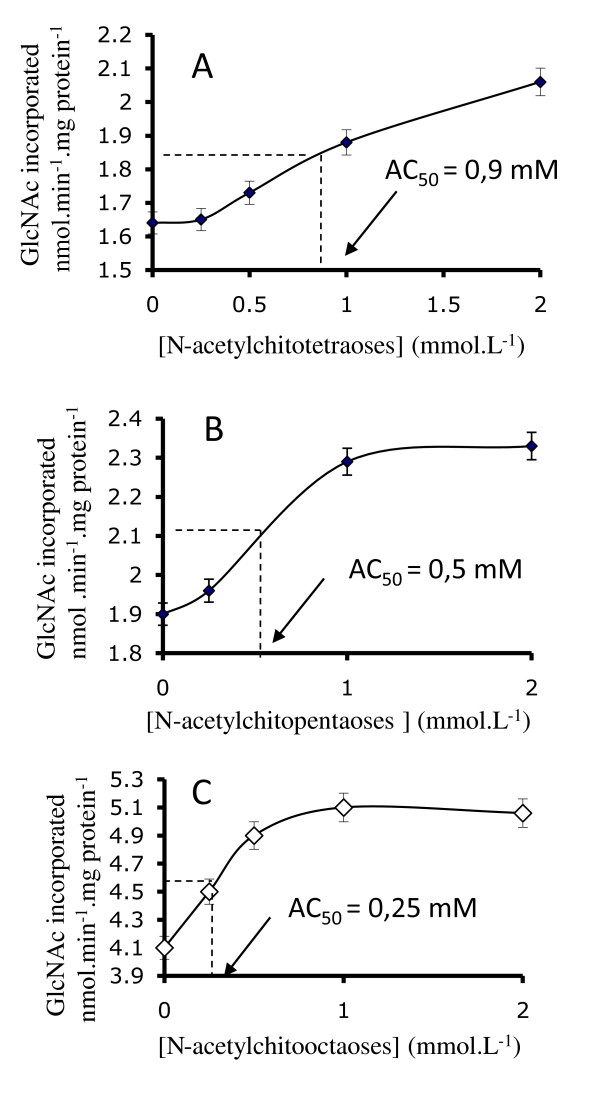
**Effect of *N*-acetylchitoose concentration on GlcNAc incorporation**. Different concentrations of *N*-acetylchito-tetra (A), -penta (B), -octaoses (C) were preincubated with membrane fractions before initiating the CHS reaction. GlcNAc incorporation into chitin versus *N*-acetylchitoose concentration is shown. AC_50 _corresponds to half-activating *N*-acetylchitoose concentration.

**Figure 5:**
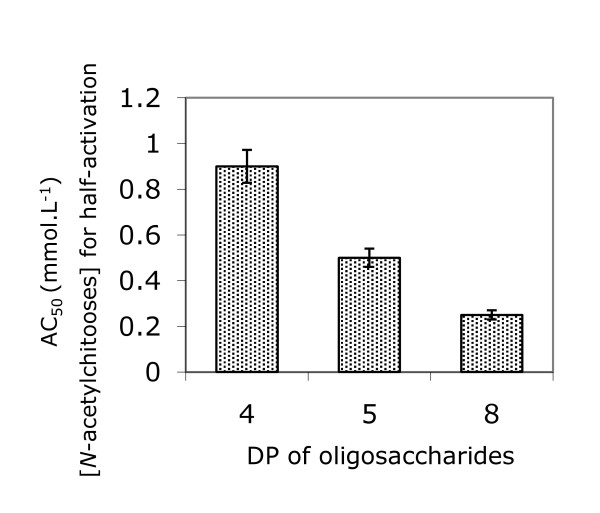
***N*-acetylchitooses units number influence half-activation concentration**. Half-activating ***N***-acetylchitoose concentration was determined from Fig. 4 for ***N***-acetylchito-tetra, -penta and -octaoses. DP = degree of polymerization.

The chitin synthesis activation effect was studied over a range of oligosaccharide concentrations from 0 to 2 mM with the *N*-acetylchito-tetra, -penta and -octaoses. It appeared that activation was a saturable phenomenon for the three *N*-acetylchitooses tested, but saturation was more clearly established with higher *N*-acetylchitoose DP (Figure 4). In each case we determined the concentration necessary to reach half-maximum activation (AC_50_) and concluded that AC_50 _values decreased as the oligosaccharide sizes increased. AC_50 _were 0.9, 0.5 and 0.25 mM for *N*-acetylchito-tetra, *-*penta and -octaoses, respectively (Figure 5). Although chitin synthesis is best characterized in *S. cerevisiae*, the mechanism of chitin biosynthesis activation or regulation is still unknown [26,25]. Three CHS isoenzymes are responsible for chitin synthesis and additional CHS proteins were identified as regulators of CHS in *S. cerevisiae *[26]. It is plausible that triggering of chitin synthesis by *N*-acetylchitooses results from oligosaccharides binding a protein partner of the complex leading to CHS activation. However, the direct binding of *N*-acetylchitooses to CHS enzyme can not be excluded. Indeed, defined oligosaccharide structures and lengths are required to observe this activation, which is also concentration dependent. Chavaroche et al. proposed that the polymerization reaction is more efficient for the synthesis of heparosan chains in presence of oligosaccharide templates, because the initiation step does not take place and the elongation of the templates occurs directly [17]. All these observations reflect the filling of an increasing number of subsites constituting the acceptor binding site of CHS. This attractive hypothesis should be compared to the reaction model scheme of chitinase, for which substrate subsites were clearly identified [27]. Cloning CHS genes and expression of recombinant proteins, in the appropriate host, would be the next step to elucidate chitin synthase activation. Our preliminary results on *Botrytris cinerea *CHS, suggest that active CHS cannot be expressed in *E. coli*. The truncated recombinant CHS protein showed specific binding to UDP-GlcNAc, but was unable to exhibit chitin synthase activity [28]. The expression of full-length CHS bound to the plasmic membrane of *S. cerevisiae *seems to be more favorable or even necessary for maintenance of enzymatic activity of CHS. Use of *N*-acetylchitooses as primers for chitin chain elongation will be verified as soon as an active recombinant chitin synthase will be obtained.

## Conclusion

In this paper, we have shown that the addition of *N*-acetylchitooses to a standard chitin synthase assay resulted in an increase of initial velocity of GlcNAc unit incorporation, specifically a 25% enhancement for *N*-acetylchitooctaoses. The effect induced by *N*-acetylchitooses is different from other known activators such as trypsin and exogenous GlcNAc. The activating effect of *N*-acetylchitoses on chitin synthesis was described as a saturable phenomenon dependant of *N*-acetylchitoose structure and length.

## Competing interests

The authors declare that they have no competing interests.

## Authors' contributions

HFB performed most of the experiments, designed and coordinated the experiments, wrote the manuscript and edited the final text. AP assisted with the enzymatic analysis, AT participated in the interpretation of data and drafted portions of the manuscript. All authors read and approved the final manuscript.
